# Identification of Membrane Fouling with Greywater Filtration by Porous Membranes: Combined Effect of Membrane Pore Size and Applied Pressure

**DOI:** 10.3390/membranes14020046

**Published:** 2024-02-07

**Authors:** Hoseok Jang, Sinu Kang, Jeonghwan Kim

**Affiliations:** Department of Environmental Engineering, Program of Environmental and Polymeric Engineering, Inha University, Inha-ro 100, Michuhol-gu, Incheon 22212, Republic of Korea; reimatarael@gmail.com (H.J.); alsrms2903@gmail.com (S.K.)

**Keywords:** greywater, membrane filtration, fouling, Hermia blocking law, dead-end filtration

## Abstract

Membrane fouling caused by complex greywater synthesized by personal care products and detergents commercially available for household applications was investigated using dead-end microfiltration (MF) and analyzed systematically by a multistage Hermia blocking model as a first attempt. The highest flux decline was associated with the smallest pore size of the membrane (0.03 μm). This effectiveness was more pronounced at higher applied pressures to the membrane. A cake layer was formed on the membrane consisting mainly of silica particles present as ingredients in greywater. Although organic rejection was low by the porous MF membrane, the organic compound contributed to membrane fouling in the filtration stage. With a 0.03 μm pore size of the membrane, dominant fouling mechanisms were classified into three stages as applied pressure increased, such as complete pore blocking, intermediate pore blocking, and cake layer formation. Specifically, during the early stage of membrane filtration at 1.5 bar, membrane fouling was determined by complete pore blocking in the 0.10 μm pore size of the membrane. However, the later stage of membrane fouling was controlled mainly by intermediate pore blocking. Regardless of the applied pressure, pore constriction or standard blocking played an important role in the fouling rate with a 0.45 μm pore size of the membrane. Our results also support that complex formation can occur due to the concentration of organic and inorganic species present in simulated greywater. Thus, strategic approaches such as periodic, chemically enhanced backwashing need to be developed and tailored to remove both organic and inorganic fouling from MF membranes treating greywater.

## 1. Introduction

The average amount of household water produced has increased worldwide owing to rapid urbanization and population growth. Therefore, the need to incorporate wastewater reuse in urban contexts for decentralized wastewater management is rapidly growing. Greywater originates from various household applications, such as bathrooms and washing machines, and is a local and important water source [1,2,3]. Greywater accounts for 30–70% of residential wastewater and often contains complex impurities depending upon the source of contaminants, such as colloidal materials, surfactants, ionic species, pathogens, and micropollutants [2,3,4,5]. Therefore, greywater treatment systems must be site-specific and water-efficient.

Membrane technology offers great potential for increasing the value of reused greywater [6]. The membrane process can meet multiple water quality criteria at a smaller footprint than other conventional wastewater treatment processes. Most importantly, the choice of membrane processes can be tailored to specific reuse applications based on the physicochemical properties of the membrane materials and the characteristics of the feed wastewater [6,7,8]. Nevertheless, membrane fouling caused by the deposition of various foulant materials present in greywater on the membrane surface or within membrane pores is a major hurdle that needs to be resolved. Membrane fouling reduces membrane permeability and rejection efficiency, thereby incurring high operational costs [9,10].

Greywater is treated directly by applying nanofiltration (NF) or reverse osmosis (RO) membranes to effectively remove cationic species and/or humic substances and meet the quality requirements for water reuse [11,12]. Nevertheless, high energy consumption and fouling potential are major concerns unless pretreatments are performed in advance. Recently, there has been an upsurge of interest in membrane bioreactors (MBRs) that combine membrane filtration with bioreactors as a pretreatment for high-pressure-driven membrane processes such as NF or RO for greywater reuse [13,14]. However, the biodegradability and toxicity of various organic compounds, such as surfactants, present in greywater are major concerns. Furthermore, the aeration required in an MBR to supply oxygen and reduce membrane fouling requires high operational energy.

Microfiltration (MF) is a low-pressure-driven membrane process that effectively removes particulate pollutants from various wastewaters. Most MF processes are carried out in dead-end filtration mode. Thus, the mass of the fouling layer will grow until all the foulants are deposited or the capacity of filter volume is reached unless periodic backwashing is performed. The MF membrane can also be applied as a pretreatment for NF or RO in membrane-integrated processes. Since the MF membrane process produces high permeate productivity per unit membrane area, it has also been widely applied to MBR systems to completely retain agglomerated biomass, thus facilitating the biodegradation of xenobiotic organic compounds. Since there are multiple applications using MF membranes for wastewater treatment, the various fouling potentials of greywater resulting from membrane pore size and operational conditions, such as applied pressure, must be better understood for membrane-based decentralized wastewater management [7,15,16,17].

Although greywater is a valuable water source, most previous studies have used simple greywater comprising a surfactant only to investigate membrane fouling. It was found that the applied pressure, feed temperature [9], surfactant concentration [12], membrane hydrophilicity, and membrane pore size affected the fouling rate and rejection efficiency [18]. Anionic surfactants such as sodium dodecyl sulfate (SDS) caused membrane fouling more than other cationic or nonionic surfactants [19]. However, analyzing fouling mechanisms in depth by MF membrane has not been attempted yet by using more complex greywater. Pidou et al. reported that the fouling rate varied depending on particle size distribution, the type of detergent used in greywater, and its composition [15]. However, the greywater prepared by adding diverse personal care products and detergents, such as shampoos, body washes, and toothpaste, should contain various organic and inorganic species, flavoring agents, abrasives, and preservatives, thus possibly complicating the fouling analysis.

Several empirical models have been applied to identify the dominant fouling mechanism in porous MF membranes. Numerous empirical models describe the Hermia blocking model to determine the dominant fouling mechanism by observing the transient behavior of the permeate flux at a constantly applied pressure [20]. This semiempirical parametric model describes four blocking laws: pore constriction or standard blocking, complete blocking, intermediate blocking, and cake layer formation [21,22,23]. The dominant fouling mechanism can be strongly time-dependent, regardless of the operational conditions, such as crossflow velocity or applied pressure [10]. However, these efforts should not be sufficient to understand the fouling mechanisms resulting from complex greywater containing diverse commercial ingredients. Therefore, this study aimed to investigate membrane fouling using porous MF membranes for treating simulated greywater in household applications. The fouling mechanism was analyzed by combining the permeate flux change with that obtained using the Hermia blocking models with respect to the membrane pore size and applied pressure in dead-end filtration mode. A better understanding of fouling mechanisms from this study will lead to the development and optimization of a cleaning strategy to reduce membrane fouling in MF membranes for greywater treatment.

## 2. Materials and Methods

### 2.1. Preparation of Simulated Greywater

**Figure A1:**
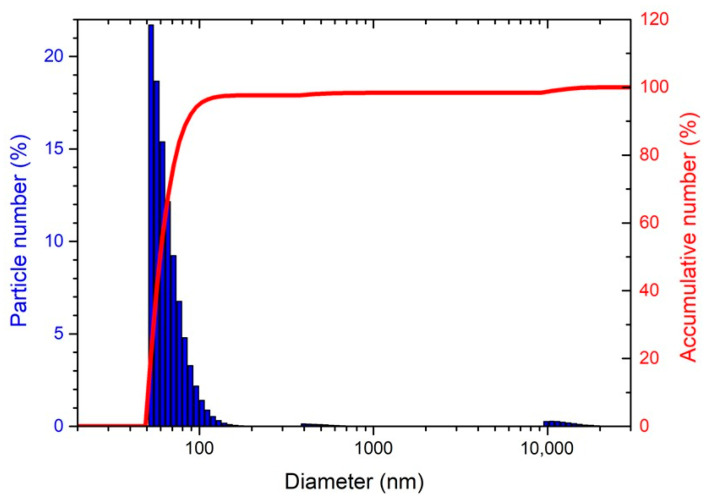
Feed greywater particle size distribution.

In this study, greywater was prepared by adding a stock solution comprising laundry detergent, shower gel, dishwasher, shaving cream, and toothpaste, which are commercially available, to reach 500 mg/L of total organic carbon (TOC). Sodium acetate, potassium phosphate, anhydrous sodium sulfate, calcium chloride dihydrate, and sodium chloride were added to the stock solution to simulate complex greywater. The solution pH was adjusted to 7.6 ± 0.3. The stock solution was diluted to 100 mg/L of TOC prior to membrane filtration. The suspended solid and total dissolved solid concentrations were 15.5 ± 3 mg/L and 444.05 ± 7.6 mg/L, respectively. The average particle size of the simulated greywater was 0.06 μm (Figure A1). The composition and characteristics of the greywater used as feed solution in this study are listed in Table 1 [24]. The experiments were performed within 1 day after preparing the stock solution to prevent particle aggregation in feed greywater solution.

### 2.2. Experimental Set-Up and Operation for Dead-End Filtration

**Figure 1:**
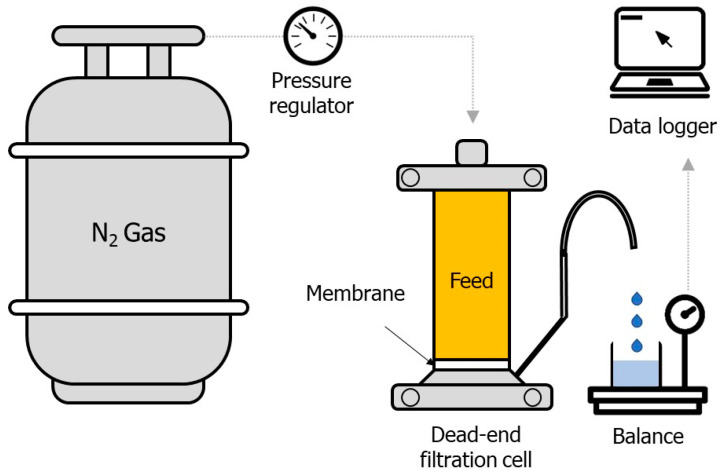
Experimental set-up of dead-end filtration cell.

A laboratory-scale dead-end filtration cell (HP4750, Sterlitech, Auburn, WA, USA) was prepared and operated at constant pressure mode with simulated greywater, as illustrated in Figure 1. A circular type of hydrophilic polyethersulfone (PES) membrane with 0.00146 m^2^ of effective surface area was equipped into the dead-end filtration cell and filtered by 300 mL of deionized (DI) water at an applied pressure of 2 bar to prevent pressure loss owing to membrane compression. A nitrogen gas tank was used to provide pressure into pressurized vessel connected to the membrane filtration cell. In this study, three different pressures (0.5, 1.0, and 1.5 bar) were applied to the dead-end filtration cell. Three different membranes with nominal pore sizes (0.03, 0.10, and 0.45 μm) were also tested (PES0034700, PES014700, PES4547100, Sterlitech, Auburn, WA, USA). Fouling rate was compared to each membrane after 300 mL of greywater filtration. The permeate produced by membrane filtration was weighed by using an electric balance located on permeate reservoir. The permeate weight accumulated in the permeate reservoir was recorded every 5 s by using a data acquisition system connected to an electronic balance (Fz-5000i A&D Company, Tokyo, Japan). The change in permeate weight was converted into permeate volume, assuming 1 g/cm^3^ of water density, to estimate permeate flux change with time. The fouling rates obtained by membrane filtration at different operational conditions were analyzed by investigating permeate flux and normalized permeability, which change with time, as shown in Equations (1) and (2):

(1) $$Permeate\;flux\;(J)=\frac{Q_{p}}{A_{m}}$$

(2) $$Normalized\;permeability\left(L_{p}\right)=\frac{J/P}{J_{0}/P}$$

where J and J_0_ are the permeate fluxes (L/m^2^/h) of the greywater and deionized (DI) water, respectively. P denotes the applied pressure (bar). Q_p_ and A_m_ are the permeate flow rate (L/h) and effective area of the membrane surface (m^2^), respectively.

### 2.3. Characterization and Analysis

Concentration of TOC and conductivity of feed greywater and permeate produced by the membrane in dead-end filtration were measured with a TOC analyzer (TOC-L, Shimadzu, Japan) based upon nonpurgeable organic carbon method and a conductivity meter (Orion Star A212, Thermo Fisher Scientific, Waltham, MA, USA), respectively. The TOC and conductivity measurements were performed within 1 h after finishing each experiment. To measure the ionic species of feed greywater and the permeate filtered through the membrane at 1.0 bar of applied pressure, ion chromatography (ICS-300, Dionex, Seoul, Republic of Korea) and inductively coupled plasma optical emission spectrometry (Optima 7300 DV, PerkinElmer, Shelton, CT, USA) were used for the cationic and anionic species, respectively. The membrane rejection was estimated using Equation (3).

(3) $$Rejection\left(\%\right)=(1-\frac{C_{p}}{C_{f}})\times 100$$

where C_f_ and C_p_ are the feed and permeate concentrations, respectively.

A Fourier-transform infrared (FTIR) vacuum spectrometer (Vertex 80 V, Bruker, Billerica, MA, USA) with the KBr pellet method was used to observe the functional groups of organics on the membrane surface and the fouling layer that formed on it. High-resolution scanning electron microscopy with energy-dispersive X-ray spectroscopy (SEM-EDX) was used to investigate the morphology of fouling layer formed on the membrane surface and its morphological characteristics. Fouled membranes were dried for 24 h at 60 °C in a dry oven and then stored in a desiccator prior to conducting FTIR and SEM-EDX analysis. To observe cross-sectional views of membranes, they were immersed in liquid nitrogen and cut carefully with a diamond knife. The particle size distribution and zeta potential were measured using a zeta potential particle size analyzer (ELS-Z, Otsuka Electronics, Kyoto, Japan) without pH adjustment. All experiments were performed immediately after taking samples.

### 2.4. Hermia Blocking Law

In this study, the Hermia blocking law was applied to know the dominant fouling mechanisms through the MF membranes treating simulated greywater. To assess fouling mechanisms involved, several assumptions were made in this study. The Hermia blocking law can be used to elucidate the fouling mechanisms during filtration stage by fitting accuracy [25,26]. Additionally, these multistage fouling phenomena relate to the types of foulant materials present in greywater. Based on the Hermia blocking law, the pattern of permeate flux change with filtration time was determined by four fouling mechanisms: pore constriction (or standard pore blocking), complete pore blocking, intermediate pore blocking, and cake layer formation. The model equations for the Hermia blocking law for each fouling mechanism are listed in Table 2. Here, K_pc_, K_cb_, K_ib_, and K_cf_ are the blocking law constants corresponding to pore constriction, complete pore blocking, intermediate pore blocking, and cake formation, respectively.

Pore constriction or standard blocking describes membrane fouling due to a reduction in membrane pore size and the deposition of foulants through internal membrane pore structures. Complete blocking can occur dominantly as single foulant settles down on a membrane pore entrance, also called “membrane sealing” [25]. For the analysis of fouling with simulated greywater using blocking law, it was assumed that the foulants block pore entrance without their superposition on membrane surface [20]. However, intermediate blocking should be driven mainly by the transfer of multiple foulants toward the membrane pore entrance through water channels. This phenomenon can occur particularly when pore entrance of membrane is deposited by foulants [20]. Finally, cake layer can be formed since the foulants are accumulated on the membrane surface [10,27].

To determine the dominant fouling mechanism, permeate flux was converted into a linearized form, for example, 1/J^0.5^, ln J, 1/J, and 1/J^2^, indicating pore constriction, complete blocking, intermediate blocking, and cake layer formation model, respectively, as shown in Table 2. Blocking model with more linearity suggests more predominant fouling mechanism. As a single fouling mechanism could not be representative of explaining whether membrane fouling occurred in entire filtration stage, a multistage Hermia model was used [10,26,28]. If the linearity is not over 0.99, it can be assumed that dominant fouling mechanism could be changed depending on elapsed filtration time. In that case, a specific filtration time to start losing linearity was investigated and considered a boundary to divide dominant fouling mechanism. Blocking law constant was optimized by fitting permeate flux data obtained experimentally through the least square method using MatLab software (R2022a, Mathworks, Natick, MA, USA) [22], as shown in Equation (4).

(4) $$Least\;square=\sum {\left(J_{p,exp}-J_{p,sim}\right)}^{2}$$

where J_p,exp_ and J_p,sim_ are the observed and simulated permeate fluxes, respectively, using each blocking law. After the blocking law constant was optimized, the permeate flux simulated was evaluated by using the coefficient of determination (R^2^) and root-mean-squared error (RMSE), as shown in Equation (5).

(5) $$RMSE=\sqrt{\frac{1}{n}\sum_{i=1}^{n}{{(J}_{p,exp}-J_{p,sim})}^{2}}$$

**Figure 2:**
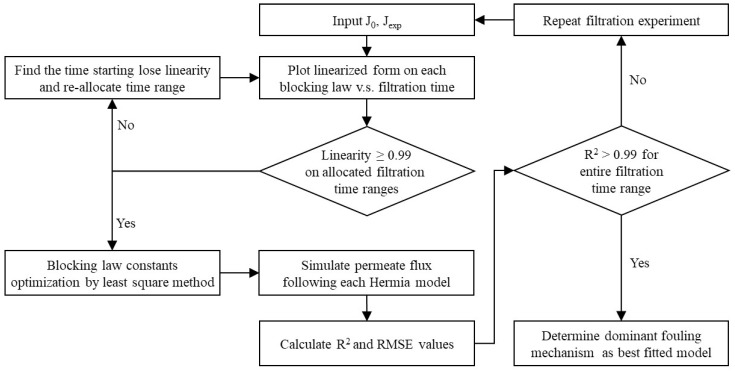
Flowchart to determine the dominant fouling mechanisms.

The procedure to determine dominant fouling mechanism is suggested in Figure 2, as below [22,23,29].

## 3. Results and Discussion

### 3.1. Combined Effect of Membrane Pore Size and Applied Pressure

**Figure 3:**
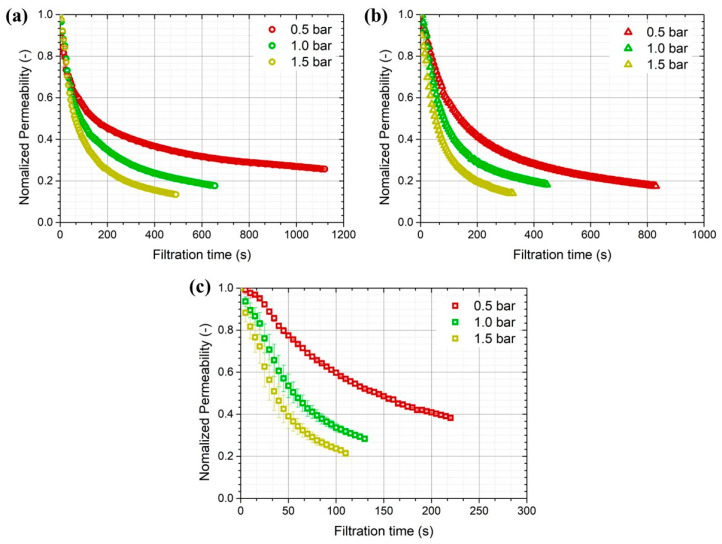
Combined effect of membrane pore size and applied pressure on flux decline with filtration time for porous membranes with (**a**) 0.03, (**b**) 0.10, and (**c**) 0.45 μm pore sizes (experiments were performed three times).

Figure 3 shows the transient behavior of the permeate flux with respect to the membrane pore size and applied pressure observed in dead-end filtration with 300 mL of simulated greywater. A higher flux decline was associated with higher applied pressure, as expected. However, when the applied pressure was constant, the rate of flux decline decreased as the membrane pore size increased. For a membrane pore size of 0.03 μm, approximately 75, 82, and 87% in flux decline was observed at applied pressures of 0.5, 1.0, and 1.5 bar, respectively. For a membrane pore size of 0.45 μm, approximately 62, 72, and 79% in flux decline was observed after filtering the same volume of simulated greywater. Flux decreased rapidly during the initial stage of membrane filtration, after which the flux gradually declined. A higher rejection of contaminants was observed with smaller membrane pore sizes, causing the development of a cake layer on the membrane surface to provide far more water transport resistance than the underlying membrane [27,30].

**Figure A2:**
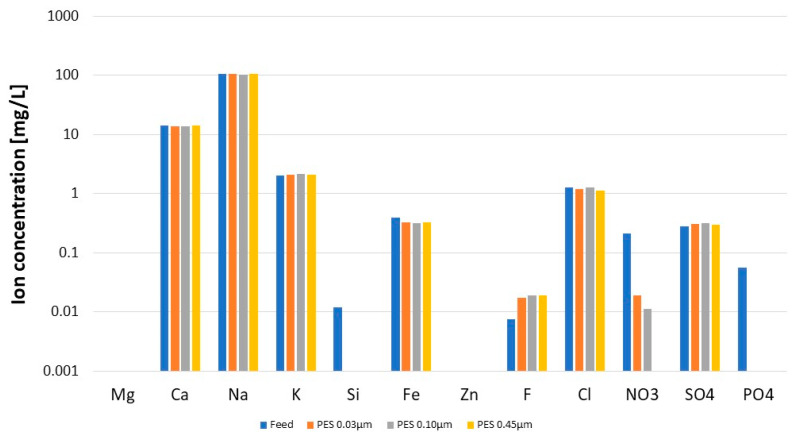
Ionic composition in feed and permeates.

Organic rejection based on TOC measurement of feed greywater and permeate was 17, 13, and 6.3% only at 0.5 bar for 0.03, 0.10, and 0.45 μm pore sizes, respectively. The rejection of inorganic species was almost negligible in this study, indicating that the inorganic species themselves present in the greywater may not contribute significantly to membrane fouling (Figure A2). From our SEM-EDX observations of a fouled membrane taken at the end of filtration, severe deposits consisting of silica particles on the membrane were observed. Silica particles in toothpaste are often used as an abrasive to clean teeth, as indicated by the stated ingredients.

Based on the analysis of variance (ANOVA), a quadratic model and a linear model were suggested to estimate membrane permeability and organic rejection, respectively, as shown in Table A1 and Table A2. The results indicated that membrane pore size was the most effective factor in determining membrane permeability and organic rejection (*p* < 0.01). The interaction parameter between membrane pore size and applied pressure was not significant compared with its individual effect. The interaction between them on organic rejection was not found either, suggesting that organic rejection could be determined more dominantly by membrane pore size or applied pressure as a single, independent effect rather than their combined effect. Since dead-end filtration was employed to treat the limited volume of synthetic greywater (300 mL) without periodic backwashing, long-term operation under periodic backwashing was not considered in this study. Further research to explain multistage membrane fouling resulting from longer filtration times between backwashing events in greywater treatment is required in future works.

### 3.2. Fouling Mechanisms of MF Membrane Filtration with Greywater

**Figure 4:**
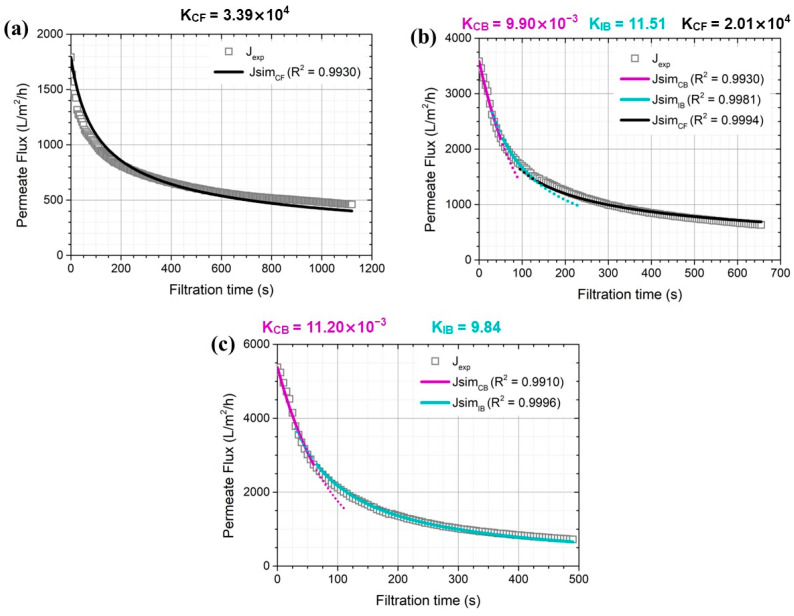
Dominant greywater fouling mechanisms on 0.03 μm membranes under different constant pressures of (**a**) 0.5, (**b**) 1.0, and (**c**) 1.5 bar.

A quantitative analysis of the transient behavior of the permeate flux measured experimentally was performed by conducting best fitting with the Hermia model. Figure 4 shows the blocking coefficient and coefficient of determination (R^2^) for a 0.03 μm membrane pore size to determine each dominant fouling mechanism based on the permeate flux change with filtration time. For the membrane pore size of 0.03 μm, membrane fouling was determined mainly by cake layer formation during the entire filtration period (K_CF_ = 3.39 × 10^4^) under 0.5 bar of applied pressure, as shown in Figure 4a.

However, increasing the applied pressure to 1.0 bar changed the dominant fouling mechanisms into three stages: complete pore blockage, intermediate pore blockage, and cake layer formation (Figure 4b). During the initial stage of membrane filtration (up to 50 s of filtration time), the permeate flux decline observed experimentally agreed well with predictions by the complete pore blocking model with an R^2^ value of 0.9930 (K_CB_ = 9.90 × 10^−3^). Membrane fouling was then dominated by intermediate pore blockage, followed by cake layer formation, with R^2^ values of 0.9981 and 0.9994, respectively. Cake layer formation occurred predominantly when the filtration time was longer than 130 s. Similar results were observed as the applied pressure increased to 1.0 bar. However, the cake layer formation coefficient (K_CF_) was relatively smaller (2.01 × 10^4^) than that obtained at an applied pressure of 0.5 bar (3.39 × 10^4^).

Considering the averaged particle size measured by synthetic greywater (0.06 μm), as shown in Figure A1, it can be suggested that the variation in permeate flux with time includes two filtration stages. Firstly, the stage is rapid flux decline within initial moments of membrane filtration because the foulants block the pore entrance without penetrating inside membrane pores [9,10,18]. As more foulants penetrate through membrane pores, the second stage starts as the number of opened pores reduced rapidly (until 50 s), leading to complete pore blocking (Figure 4c). As applied pressure to the membrane became lower, however, fewer foulants should enter the membrane pores, probably due to the smaller drag force acting on the foulants [10]. These results support the fact that cake layer formation determines the fouling rate more dominantly at relatively lower applied pressure into the MF membrane [18,30].

**Figure 5:**
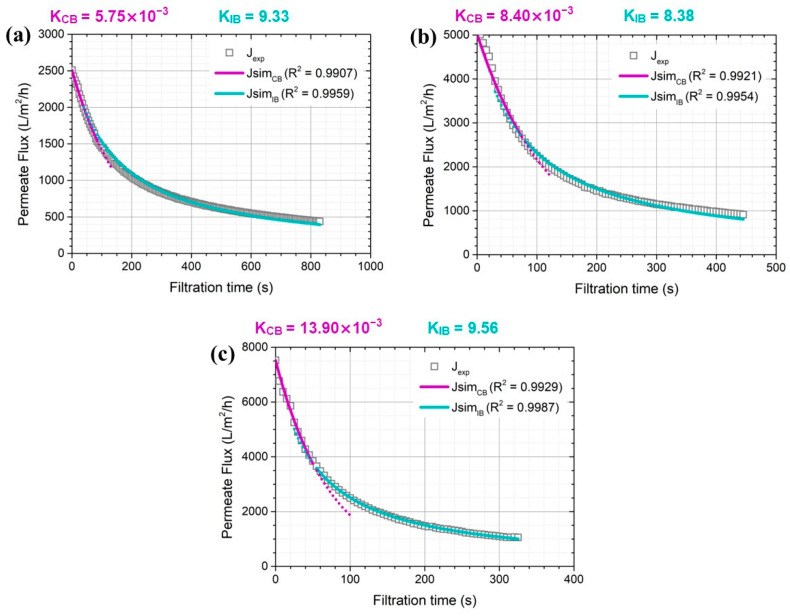
Dominant greywater fouling mechanisms on 0.10 μm membranes under different constant pressures of (**a**) 0.5, (**b**) 1.0, and (**c**) 1.5 bar.

For the 0.10 μm membrane pore size, fouling mechanisms were controlled by two successive stages regardless of applied pressures and complete and intermediate blocking. Permeate flux decreased rapidly until about 85 s of filtration time due to complete pore blocking by yielding R^2^ values of 0.9907, 0.9921, and 0.9929 at applied pressures of 0.5, 1.0, and 1.5 bar, respectively (Figure 5). With a large ratio of nominal membrane pore size to particle size (0.10 vs. 0.06 μm), there should be more chance that each solute in greywater contributes to pore blocking by means of pore sealing without their superposition. When the filtration time is longer than 85 s, each solute in greywater can be settled down on the membrane surface or on the solute previously arrived so that it can seal the membrane pore and then move forward to intermediate blocking [10,28].

**Figure 6:**
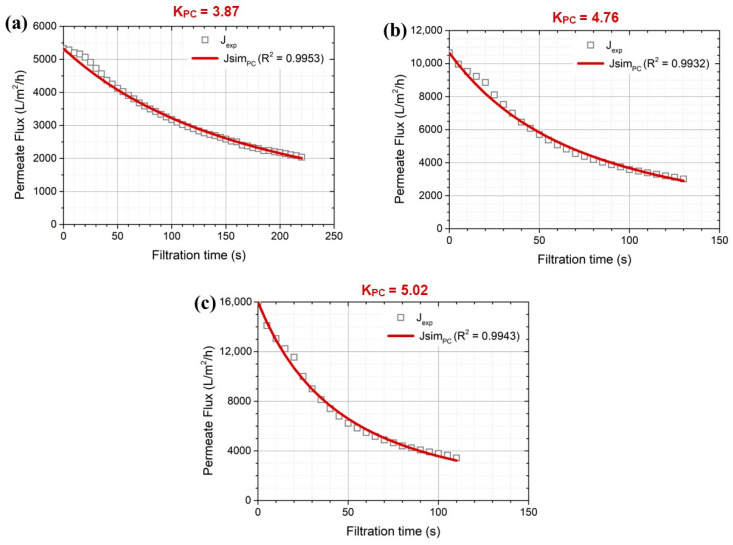
Dominant greywater fouling mechanisms on 0.45 μm membranes under different constant pressures of (**a**) 0.5, (**b**) 1.0, and (**c**) 1.5 bar.

For the 0.45 μm membrane, which was the largest pore size used in this study, pore constriction dominated the fouling rate during the entire filtration period (Figure 6). A higher pore constriction coefficient was associated with higher applied pressures, showing 3.87, 4.76, and 5.02 K_pc_ at 0.5, 1.0, and 1.5 bar, respectively. With porous membrane filtration, pore constriction can occur because pore volume reduces gradually due to the adsorption and deposition of solutes on the pore walls. Pore volume can also be reduced due to the volumetric accumulation of solutes within a membrane. It is known that this blocking mechanism should become more pronounced with larger membrane pore size, especially for the treatment of dilute solution containing solutes such as colloidal materials [28], organic compounds [26,29,31], and surface water containing natural organic matter (NOM) [21,22,32]. Thus, a strategic approach is needed to mitigate membrane fouling in greywater filtration using MF membranes depending upon different filtration stages; for example, periodic chemically enhanced backwashing tailored to remove fouling resistance needs to be optimized.

Table 3 summarizes the results obtained by using Hermia model analysis with respect to membrane pore size and applied pressure observed in this study. The values of the simulated permeate flux fitted well with the flux obtained by performing dead-end filtration experimentally. Fouling mechanisms in greywater filtration using the MF membrane were significantly affected by membrane pore size and applied pressure. Clearly, the cake layer formation was an increasingly dominant fouling mechanism as the membrane pore size reduced. Irrespective of applied pressure, the contribution of pore constriction (standard blocking) to the fouling rate was greater with the larger pore size of the membrane.

### 3.3. Membrane Rejections and Microscopic Observations

**Figure 7:**
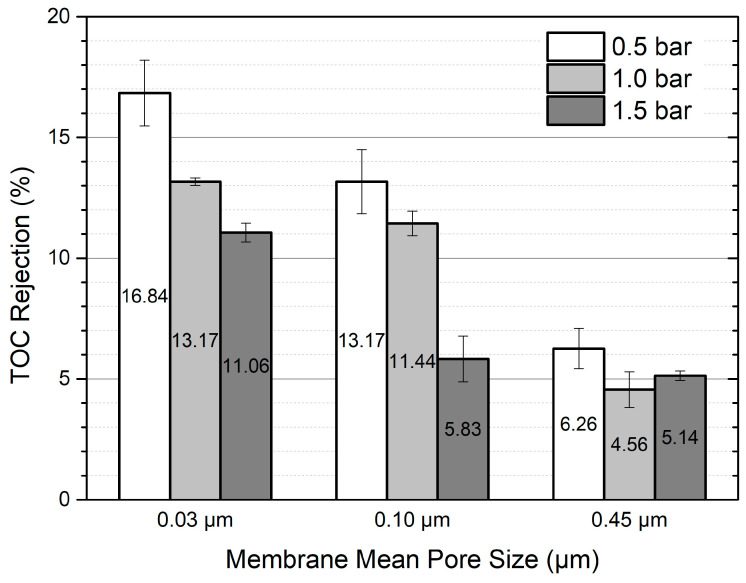
Total organic carbon rejection after filtration with 300 mL of greywater.

Rejection was quantified by the total organic carbon (TOC) and suspended solids (SSs) filtered through different membrane pore sizes and applied pressures. Figure 7 shows that less organic rejection is associated with a larger pore size of the membrane, as expected. However, only 17% of TOC rejection was observed as the maximum value of the 0.03 μm pore size of the membrane was used. The SS concentration was not detected in the permeate after membrane filtration. Irrespective of the membrane pore size and applied pressure, the rejection of total dissolved solids (TDSs) was only less than 3%.

**Figure A3:**
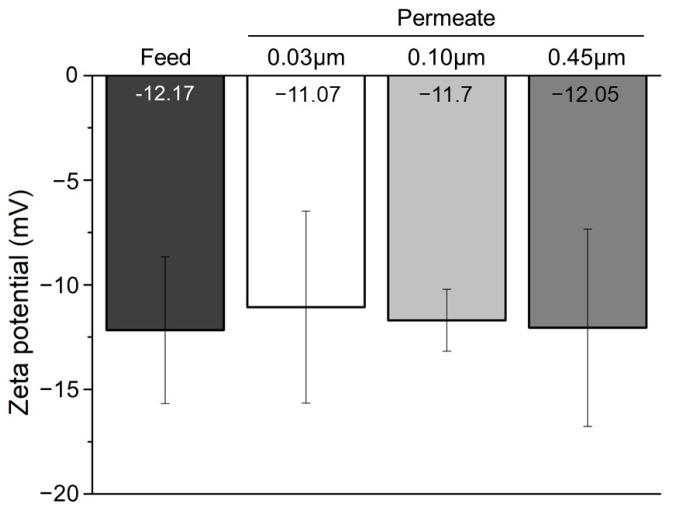
Solution zeta potential for feed and permeates depending on membrane pore size at 0.5 bar filtration.

Zeta potentials were also measured in the feed greywater and the permeate by each membrane filtered at 1 bar of applied pressure (Figure A3). After filtration, there was no significant change in zeta potential showing −12.17, −11.07, −11.7, and −12.05 mV for feed greywater, and the permeate obtained from 0.03 μm, 0.10 μm and 0.45 μm membrane, respectively. The surface zeta potential of the clean and fouled membranes (0.03 μm pore size) measured at 0.5 bar was −26.8 and −25.6 mV, respectively. Our results strongly indicate that electrostatic interaction between charged foulants and the membrane should almost be negligible to determine the fouling rate based on our short-term operation performed by dead-end filtration. Although the permeate flux was enhanced by increasing applied pressure, TOC rejection was lower because more organics could pass through the membrane [30]. Although only a small fraction of TOC is rejected by the MF membrane, organic compounds are also very likely to cause membrane fouling in greywater treatment, causing flux decline. For example, surfactants, linear alkyl benzene, and SDSs in a TOC fraction were important organic foulants in greywater in spite of their low rejection [12,19]. In a study of membrane fouling by greywater, xenobiotic organic compounds (XOCs) in household chemical products, such as bleaches, softeners, and builders, were found to reinforce the organic fouling [4,5,33]. It has also been reported that cationic surfactants or nonionic surfactants are more responsible for fouling resistance than those caused by anionic surfactants [19]. For anionic surfactants, the rejection of TOC through a hydrophilic ceramic membrane with a 0.05 μm pore size was only 5% [34]. Nevertheless, this rejection was improved (about 50%) by using the small pore size of a ceramic membrane (0.02 μm) operated by crossflow filtration [35]. A previous study also reported that only 5% of TOC could be rejected by 0.22 μm hydrophilized PVDF membrane under dead-end filtration while causing significant flux decline [19]. Since these studies used simple greywater, for example, anionic surfactant alone, and the operational mode was different, it might be difficult to make a direct comparison and draw conclusions on organic rejection with complex greywater. Nevertheless, fouling resistance due to the organic materials occurred undoubtedly after greywater filtration through the MF membrane.

**Figure 8:**
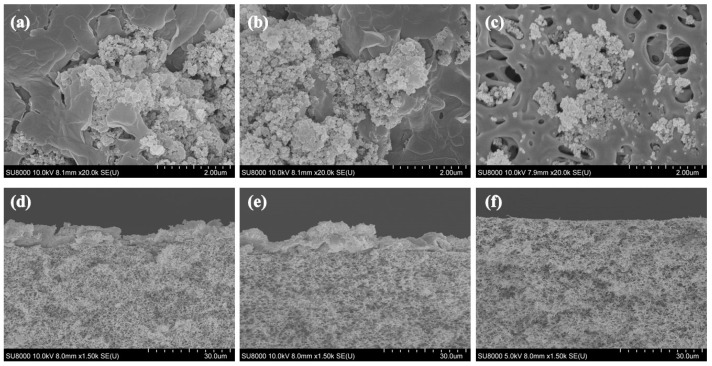
SEM images on fouled membrane surfaces with pore sizes of (**a**) 0.03, (**b**) 0.10, and (**c**) 0.45 μm and cross-sections of membranes with pore sizes of (**d**) 0.03, (**e**) 0.10, and (**f**) 0.45 μm after 300 mL greywater filtration at 1.0 bar pressure.

SEM images of the fouled membrane captured at the end of membrane filtration are presented in Figure 8. The relative extent of membrane fouling that accumulated on the membrane surface became more severe as the membrane pore sizes became smaller, as discussed above. With a membrane pore size of 0.45 μm, no evidence of total pore blocking was observed by SEM. However, in the SEM image, there is considerable evidence of an external fouling layer or cake layer as an agglomerated form on membranes with pore sizes of 0.03 or 0.10 μm.

**Figure 9:**
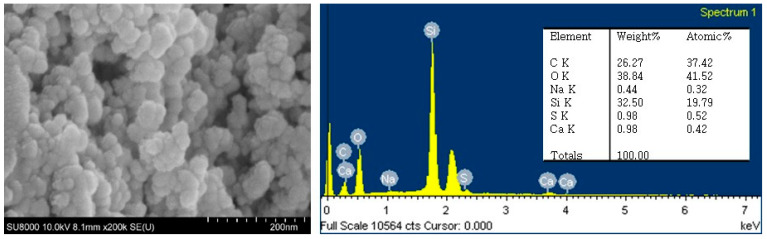
SEM-EDX results of grape-like shape particulates on the fouled membrane surface.

The SEM-EDX observations with the external fouling layer indicated that the most prevalent inorganic particle deposited on the membrane surface was Si (19.8%; Figure 9). The presence of Si originated from the nanosilica particles in the toothpaste used as the stated ingredient for polishing in greywater.

With greywater filtration, complex formation between organic compounds and cationic species may also lead to an aggregated form of the fouling layer, causing pore blocking or a dense cake layer. The simulated greywater prepared in this study contained high amounts of cations, such as calcium (10 mg/L), sodium (100 mg/L), and ferrous ions (1 mg/L) as measured in this study. Colloidal systems containing lower calcium concentrations cause smaller aggregated forms, which can cause pore blocking within membrane pores or a dense cake layer formation on the surface of a membrane [36]. However, increasing the calcium concentration facilitates the formation of porous aggregates that are interconnected loosely [37]. This fouling layer has a lower fouling resistance against water transport than that caused by a dense cake layer [38]. In the porous aggregate form of the fouling layer, the retention of both organic compounds and cations in greywater can be enhanced by secondary filtration. Our results also showed that greywater had great potential to cause pore blockage or dense fouling layers with aggregated complex forms. Furthermore, interconnected foulants can clog the membrane pore entrance so that they can pass through the membrane more easily as the applied pressure increases. Meanwhile, complete blocking was not disclosed early as the applied pressure was reduced to 0.5 bar, showing an R^2^ value of 0.886.

**Figure 10:**
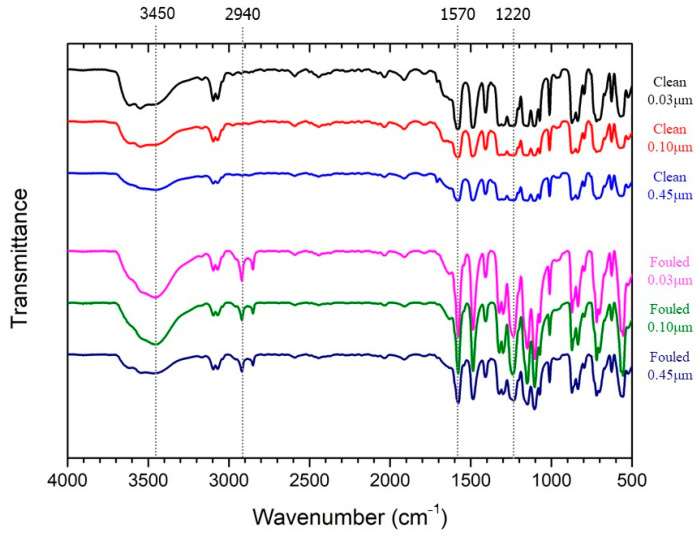
KBr-FTIR spectra on the clean and fouled membrane surfaces.

The FT-IR spectra of the cleaned and fouled membrane surfaces showed five peaks at 3450 cm^−1^ (stretching OH), 2940 cm^−1^ (aliphatic-CH_2_ stretching), 1570 cm^−1^ (stretching C-N and bending NH), and 1220 cm^−1^ (stretching C-O and OH deformation of COOH), as shown in Figure 10. Each peak suggests the presence of lipids, proteins, and polysaccharides, respectively [39,40]. Peak A, which is an intense band of polysaccharides, was observed on membranes with pore sizes of 0.03 and 0.10 μm. However, Peak B, corresponding to lipids, was found on the 0.45 μm pore size of the membrane. This difference clearly indicates that most organics, comprising proteins and polysaccharides, should pass through a 0.45 μm membrane pore size.

## 4. Conclusions

The Hermia blocking model was applied to evaluate the dominant fouling mechanisms by the MF membrane treating simulated complex greywater. The transient behavior of the permeate flux decline through dead-end filtration agreed well with that predicted by the Hermia blocking model. Complete pore blockage could not be avoided during the initial stage of membrane filtration for any combination of pore size of the membrane (0.03 and 0.10 μm) and applied pressure. With filtration time, complete pore blocking was transferred into intermediate pore blocking. Reducing the membrane pore size accelerated cake layer formation, particularly at the lowest applied pressures (0.5 bar). Regardless of the applied pressure, however, pore constriction or standard blocking was dominant in determining the fouling rate, particularly for a larger pore size of the membrane (0.45 μm). Microscopic observations of the membrane surface suggested that both inorganic particles, such as silica and organic compounds, are important foulants in greywater filtration. Moreover, the accumulation of organic compounds and inorganic particles as agglomerates forming over filtration time suggests that complexes should be formed by greywater filtration, and they could be attached to the membrane. Fouling control in greywater treatment by porous membranes is important to reduce the operational burdens of membrane systems. Nevertheless, since greywater remains a mainly underutilized resource, it could be reused after coupling greywater membrane treatment systems with further treatment to produce reusable clean water.

## Figures and Tables

**Table 1:** Simulated greywater composition and characteristics.

Item	Value
C_2_H_3_NaO_2_	384 mg/L
NH_4_Cl	83 mg/L
K_2_HPO_4_	3 mg/L
Na_2_SO_4_	51 mg/L
CaCl_2_	50 mg/L
NaCl	50 mg/L
Shampoo	53 mg/L
Shower gel	143 mg/L
Toothpaste	36 mg/L
Shaving cream	53 mg/L
Laundry detergent	208 mg/L
Dishwasher	87 mg/L
pH	7.6 ± 0.3
Total organic carbon	103 ± 2.2 mg/L
Suspended solids	15.5 ± 3 mg/L
Total dissolved solids	444.05 ± 7.6 mg/L
Mean particle size (d_50_)	59.6 nm

**Table 2:** Model equations and schematic of Hermia blocking law.

Fouling Mechanism	Equation	Linearized Equation Form	Schematic
Pore constriction (PC)	$J=\frac{4\cdot J_{0}}{{\left(K_{pc}\cdot J_{0}\cdot t+2\right)}^{2}}$	$\frac{1}{J^{0.5}}=\frac{1}{J_{0}^{0.5}}+K_{pc}\cdot t$	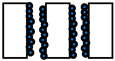
Complete blocking (CB)	$J=J_{0}\;exp\left(-K_{cb}\cdot t\right)$	$\ln\;J=ln\;J_{0}+K_{cb}\cdot t$	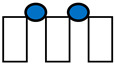
Intermediate blocking (IB)	$J=\frac{J_{0}}{K_{ib}\cdot J_{0}\cdot t+1}$	$\frac{1}{J}=\frac{1}{J_{0}}+K_{ib}\cdot t$	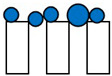
Cake formation (CF)	$J=\frac{J_{0}}{{\left({2K}_{cf}\cdot J_{0}^{2}\cdot t+1\right)}^{\frac{1}{2}}}$	$\frac{1}{J^{2}}=\frac{1}{J_{0}^{2}}+K_{cf}\cdot t$	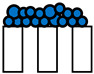

**Table 3:** Dominant fouling mechanisms and Hermia model parameters depending on the applied pressure and membrane pore size (PC: pore constriction, CB: complete blocking, IB: intermediate blocking, CF: cake formation).

Pore Size (μm)	Applied Pressure (bar)	Dominant Fouling Mechanism (Hermia Constant)					All Filtration Range	
							RMSE (as L/m^2^/h)	R^2^
0.03	0.5	CF (3.39 × 10^4^)					58.0	0.9930
	1.0	CB (9.9 × 10^−3^)	→	IB (11.51)	→	CF (2.02 × 10^4^)	41.3	0.9962
	1.5	CB (11.2 × 10^−3^)		→	IB (9.84)		58.6	0.9972
0.10	0.5	CB (5.7 × 10^−3^)		→	IB (9.33)		35.1	0.9963
	1.0	CB (8.4 × 10^−3^)		→	IB (8.38)		80.0	0.9945
	1.5	CB (13.9 × 10^−3^)		→	IB (9.56)		51.0	0.9990
0.45	0.5	PC (3.87)					94.4	0.9953
	1.0	PC (4.76)					221.8	0.9932
	1.5	PC (5.02)					282.4	0.9943

## Data Availability

The data are contained within the article.

## Appendix A

**Table A1:** Statistical analysis for normalized membrane permeability depending on pore size and applied pressure.

Y = Normalized Permeability at 300 mL Feed Filtration (−) *							
$Y=7.6+5.19A-6.92B-1.37AB+15.66A^{2}+1.34B^{2}$							
	ANOVA					Statistic	
	Sum of Squares	df	Mean Square	F-Value	*p*-Value	R^2^	Adjusted R^2^
Quadratic model	732.48	5	146.5	194.58	0.0006	0.997	0.992
A: Pore size	161.9	1	161.9	215.04	0.0007		
B: Pressure	269.9	1	269.9	358.5	0.0003		
AB	8.65	1	8.65	11.49	0.0428		
A^2^	131.81	1	131.81	175.08	0.0009		
B^2^	3.57	1	3.57	4.74	0.1177		
Residual	2.26	3	0.7529				
Cor Total	734.74	8					

* Independent variables (A: pore size and B: applied pressure) are actual values.

**Table A2:** Statistical analysis for total organic rejection depending on pore size and applied pressure.

Y = Total Organic Rejection at 300 mL Feed Filtration (%) *							
Y=8.93−3.83A−2.37B							
	ANOVA					Statistic	
	Sum of Squares	df	Mean Square	F-Value	*p*-Value	R^2^	Adjusted R^2^
Linear model	134.92	2	67.46	17.52	0.0031	0.8538	0.805
A: Pore size	101.12	1	101.12	26.26	0.0022		
B: Pressure	3.38	1	33.8	8.78	0.0252		
Residual	23.11	6	3.85				
Cor Total	158.02	8					

* Independent variables (A: pore size and B: applied pressure) are actual values.
